# Prevalence of *Lawsonia intracellularis* in pig herds in different European countries

**DOI:** 10.1186/s40813-019-0137-6

**Published:** 2019-12-17

**Authors:** Mirjam Arnold, Annelies Crienen, Hanny Swam, Stephan von Berg, Rika Jolie, Heiko Nathues

**Affiliations:** 1 Clinic for Swine, Department for Clinical Veterinary Medicine, Vetsuisse Faculty, University of Bern, Bern, Switzerland; 2 Center for Diagnostic Solutions, MSD AH Boxmeer, Boxmeer, The Netherlands; 3 MSD Animal Health, Munich, Germany; 4 Merck Animal Health, Madison, NJ 07940 USA

**Keywords:** qPCR, ELISA, Denmark, Germany, Spain, France, Netherlands, United Kingdom

## Abstract

**Background:**

*Lawsonia intracellularis* causes large economic losses in the pig industry worldwide. Pigs suffer from reduced daily weight gain, poor feed conversion ratio and increased mortality. The number of affected animals and herds in Europe remains unknown. This study will provide an overview of the prevalence of *Lawsonia intracellularis* in herds with a history of diarrhoea in different European countries and thereby identify country specific differences.

**Results:**

Out of the 144 herds sampled in Germany, Denmark, Spain, the Netherlands and the United Kingdom, 90.3% (79.2–100.0%) contained at least one positive faecal sample on quantitative polymerase chain reaction (qPCR). Of the 6450 nursery, growing and finishing pigs of the previously mentioned herds, 26.2% (15.9–41.5%) of the animals were tested positive in faecal samples. Enzyme linked immunosorbent assay (ELISA) results of 60 herds were 91.7% (70–100%) positive. The percentage of positive samples in these 1791 blood samples was 31.6% (20.3–51.0%). Herd prevalence did not differ significantly by qPCR or ELISA. Significant differences between the countries were found regarding: Within-herd prevalence- qPCR: Samples from Denmark were more often positive than samples of Spain or the United Kingdom. Within-herd prevalence- ELISA: Samples from Denmark were more often positive than samples from Spain and the Netherlands. Affected age category- qPCR: Nursery pigs in Denmark were more often positive and shed more genome equivalents than nursery pigs in the other countries. Concentration of detected genome equivalents- qPCR: The concentration of genome equivalents from *Lawsonia intracellularis* in herds in Denmark was higher compared to all other countries.

**Conclusion:**

A widespread of *Lawsonia intracellularis* in the six European countries was confirmed, whereby a large part of the positive animals only excreted small amounts of genome equivalents. Country specific differences were found with Denmark in particular diagnosing more *Lawsonia intracellularis* then the other countries. Herd data collected in this study needs to be analysed to get more information about possible reasons for the differences found between the countries.

## Background

The bacterium *Lawsonia* (L.) *intracellularis* is widespread in all pig- keeping continents worldwide [1–4]. As the cause of porcine proliferative enteropathy (PPE), it is described to have a large economic impact on the pig production system [5]. Losses due to its negative impact on daily weight gain, feed conversion ratio and mortality effects [6, 7] have been reported. These losses vary from country to country. While Germany reports profit setbacks of 1.2% /farm [7], in Denmark 1.5 to 3.0 US$ per weaner [8] and in the UK 2 to 7£ per affected fattening pig is documented [6]. Since the prevalence of infected herds and diseased animals in Europe is unknown, exact estimations of the impact in Europe are difficult. Results of peer reviewed prevalence studies in European countries since the year 2000 are listed in Table 1. Reported herd prevalence in the countries vary from 6.7 to 93.7% while the number of positive animals per herd (within-herd prevalence) ranges from 0.7 to 43.2%. For the interpretation of these values, however, the influencing factors ‘age’ [12] as wells as ‘diagnostic methods’ [16] should be taken into account. Additionally it is known, that *L. intracellularis* does not necessarily lead to disease [17]. Faecal presence of *L. intracellularis* determined by qualitative PCR does, therefore, not lead to information about clinical, subclinical or absence of infection [17]. Quantitative tests on the other hand can be used to detect the concentration of the pathogen and thus draw conclusions about an infection. The detection of antibodies, does only indicate an exposure to the pathogen in the past [18] and does not provide information about current infections in the animals.

**Table 1:** Overview of peer reviewed prevalence studies performed in Europe since the year 2000

Country	Age categories of sampled animals	Diagnostic material and method	Herd-Prevalence (%)	Within-herd-Prevalence (%)	Source
Denmark	GP, FP	Faeces: PCR	93.7	25	[9]
France	NP, GP, FP, S	Blood: IFA	88		[10]
Germany	NP, GP, FP, S	Faeces: PCR	30* 14**	19.4* 7.3**	[11]
Germany	NP, GP; FP, S, B	Blood: IFAT	81.3	43.2	[12]
Germany	SP, NP	Faeces: nPCR Blood: ELISA	6.7 39.2	0.7 5.2	[13]
Great Britain	NP, GP, FP	Blood: IFA	93.1		[14]
Republic of Ireland	NP, GP, FP	Blood: IFA	92.9		[14]
Spain	NP, GP, FP, S	Blood: IFA	68.96		[10]
Sweden	GP	Faeces and rectal swabs: nPCR	47.6	27.1	[15]

A herd was defined positive, if at least one sample was positive for *L. intracellularis* (herd- prevalence). The within- herd prevalence is the number of samples positive per analysed samples per herd within all herds per country. Age categories were classified as follows: Suckling pigs (SP) = before weaning, Nursery pigs (NP) = weaner till ~ 25 kg, Growing pigs (GP) = ~ 25- 40 kg, Finishing pigs (FP) = ~ 40 kg till slaughter, Sows (S), Boars (B)

Diagnostic methods: Enzyme linked immunosorbent essay (ELISA), indirect Immunofluorescence assay (IFA), indirect Immunofluorescence antibody test (IFAT), Polymerase chain reaction (PCR), nested PCR (nPCR)

* = animals with diarrhoea; ** = animals without diarrhoea

Faecal shedding in an experimental study was first detected one week after exposure to *L. intracellularis* [19, 20]*.* First shedding under field conditions was recorded at the age of 6 weeks in Germany [21, 22] and from approximately 8 weeks onwards in Denmark [23]. The maximum of faecal shedding is described at the age of 9 to 10 weeks [21] or 10 to 12 weeks of age [23] respectively. Shedding is intermittent, whereby the duration differs between one to eight weeks depending the source [21–23]. However, other sources report (intermittently) excretions of 10 weeks and more [19, 24, 25]. In further studies, detection of *L. intracellularis* via PCR in faeces from an age of 14 and 18 weeks, respectively, until slaughter was no longer possible [21–23].

A correlation between the determined dose of *L. intracellularis* and presence of histopathological lesions and average daily gain is known [26, 27]. In a Danish study, the median of bacteria found in animals with gross lesions (6.01 log10 bacteria/g faeces), was significantly higher compared to animals without gross lesions [26]. Moreover in an experimental study, the average daily gain reduced sharply, when shedding of *L. intracellularis* increased from 10^7^ to- 10^8^ [27]. Pigs dosed with approximately 3.7 × 10^6^ organisms developed severe lesions of PPE detected by histopathology [17]. In addition, the effects on mortality and average daily gain were greater, when a higher dose (5.4 × 10^8^ to 5.4 × 10^10^ organisms) was administered. Animals that excrete *L. intracellularis* (or GE of *L. intracellularis*) in high amounts therefore are more likely to suffer from clinical or subclinical PPE [27]. However, it is not yet possible to say definitively at what level the first impairments of the animals do occur [26].

About 2 to 6 weeks after first shedding of *L. intracellularis,* seroconversion is described [23, 24]. In contrast, also seroconversion prior to shedding is reported [21]. Due to maternal antibodies, which are detectable up to 4–5 weeks of age, seroconversion can be hidden [10, 24, 28, 29]. However, these maternal antibodies do not appear to be able to fully provide protection from infection [24], but some literature has shown the possibility of partial protection [30]. It is known that exposed sows transfer maternal antibodies, while differences per parity of the sow seem to exist [31]. In the meantime, the impact of sow herds as a source of infection for piglets is not fully understood [10, 24, 31]. Seroconversion in an experimental study was described within two weeks after exposure [19] and protective immunity was reported few weeks after infection [30, 32]. A first seroconversion of pre-negative piglets in Germany and Denmark was detected at 6 to 10 weeks of age mainly increasing after 2 to 6 weeks later [12, 21, 23], while in Spain and France first seroconversion is reported at 8 to 16 weeks of age [10].

How long animals remain seropositive is not uniformly reported. In some studies the majority of animals remained seropositive over weeks until slaughter [19, 23], or in the case of sows up to three months [28]. On the other hand also studies in which less than 50% of the animals remained seropositive until slaughter are reported [21, 22].

## Methods

The aim of this study was to provide an overview of the prevalence of *L. intracellularis* in pig herds with a history of diarrhoea in different European countries all measured by the same diagnostic methodology and in same age categories. Thereby comparison between the countries were possible and potential country-specific differences were shown.

The presence of *L. intracellularis* genome equivalents (GE) in faeces and antibodies in serum were examined in pig herds in six European countries: Germany (DE), Denmark (DK), Spain (ES), France (FR), the Netherlands (NL), and the United Kingdom (UK). From October 2017 to November 2018 faecal and blood samples were taken in 24 herds per country. Thereby sampling was mostly uniformly distributed throughout the year. Faecal samples were then analysed with a quantitative real time polymerase chain reaction (qPCR) while blood was analysed with an enzyme-linked immunosorbent assay (ELISA). Based on the diagnostic structure of the study, results were usually submitted to the farmer 3 weeks (ELISA results) or 6–8 weeks (qPCR results) after sampling took place. Furthermore, information about the farm, such as, herd structure, feeding, hygiene management and vaccination were collected in a questionnaire filled in by a veterinarian, who subsequently sampled the herd. The questionnaire was then sent to the Clinic for Swine, VETSUISSE Faculty, University of Bern, Switzerland. All herds were selected and sampled by veterinarians employed by a local branch of MSD Animal Health, taking in- and exclusion criteria into account.

### Inclusion and exclusion criteria

Only herds with at least one outbreak of diarrhoea in the preceding 12 months before the date of the examination were included. ‘Diarrhoea outbreak’ was defined as having (had) clinical signs of diarrhoea in the pig herd, according to the pig producer and/ or veterinarian. The suspected cause was not of interest. In addition, participation in the study was allowed only once. The production system had to be a farrow-to-finish farm, or nursery−/ fattening- farm, receiving all their animals from one single origin. Within four weeks prior to sampling no antimicrobial treatment was allowed, whereas vaccination against *L. intracellularis* and other pathogens was no exclusion criteria.

### Sampling procedure

Before sampling took place, faecal containers and blood tubes were labelled with the sampling date, sampling material, country, number of the herd and individual number of the sample which included the age category. Thereby blood samples could always be assigned to the corresponding faecal sample. Animals were divided into the following three categories based on their age and weight: nursery pigs with a bodyweight of approximately 10 to 25 kg; growing pigs with a bodyweight of approximately 25 to 40 kg and finishing pigs with a bodyweight of approximately 40 to 100 kg. During a random walk [33], a random selection of animals within these three categories was performed. This means that animals and pens were selected randomly while walking with a colour spray all over the compartment. The current state of health like diarrhea or normal faeces was not considered. **Faecal samples**: At least 2 g of native faeces from the rectum of 15 animals per age category and herd were sampled. To avoid cross contamination between the samples, non-sterile gloves had to be worn and changed after each animal. **Blood samples:** At least 6 mL of blood were taken from the *Vena jugularis externa*. Due to sample size calculation and economics, blood was sampled in 10 of the 15 animals per age category, and in 10 of the 24 herds per country. Sampled blood was stored overnight to clot at approximately 23 °C and was then centrifuged for 10 min at 2500 g. Subsequently the supernatant serum samples were stored at − 20 °C until they were shipped together with the cooled or frozen faecal samples in refrigerated containers to a laboratory in the Netherlands.

### Laboratory analyses: qPCR and ELISA

All faecal samples were analysed with a qPCR to detect the concentration of specific GE of *L. intracellularis*. The commercial kits ‘Kylt® PIA (*Lawsonia intracellularis*)’ and ‘Kylt® Quantitative standard for *Lawsonia intracellularis*’ from AniCon Labor GmbH, Hoeltinghausen, Germany were used according to manufacturer’s instruction. In conclusion, only curves with the typical exponential amplification were considered positive. In some samples a CT value outside the linear range (i.e. lower than the lower limit of quantification - LLQ- or higher than the upper limit of quantification - ULQ) was found. Samples on the upper part were then retested in a dilution to determine the exact concentration. Samples below the lowest standard sample were reported as `positive but below the quantification range`. In the further calculations, these samples below the limit of quantification were included with a concentration of 1 GE/ μl. There is no detailed information on sensitivity and specificity in the manuals of the commercial kit. For a better comparability with other data published in other studies, a recovery and absolute quantification experiment was carried out. Therefore a sample panel of 40 samples (high, medium, weak, around and below the limit of detection and negative samples) was divided into two sample containers per sample. One was send to the Field Station for Epidemiology, University of Veterinary Medicine, Hannover, Germany for analysis with a qPCR, whereof MIQE complying data have been published [34]. The other one was used for the extraction and analysis at MSD AH Boxmeer, Center for Diagnostic Solutions in the Netherlands. An average difference in log titres between the two assays was detected with 0.4 log. The difference was not dependent on the positivity of the sample. It did not increase significantly when the titre was lower. Both assays showed a comparable linearity and a correlation of more than 95%. It is therefore assumed, that the sensitivity and specificity of the commercial test kit is comparable to the published qPCR used in the Field Station for Epidemiology in Germany [34] to which it was compared.

All supernatant serum samples were analysed with an ELISA for *L. intracellularis* antibody detection. The commercial kit ‘SVANOVIR® L. intracellularis/ Ileitis-Ab’ from Boehringer Ingelheim SVANOVA®, Uppsala, Sweden was used. All faecal and supernatant serum samples were shipped to the MSD AH Boxmeer Center for Diagnostic Solutions in the Netherlands.

### Statistical analysis

The number of required herds per country in order to estimate the herd prevalence was calculated for an unknown population size, a confidence level of 95% and an expected prevalence of 50% with an accepted error of 20% [35]. The determined sample size needed was therefore 25. In order to consider possible seasonal effects, it was decided to sample six herds per quarter in each country. The number of samples needed per herd, in order to detect an infection with *L. intracellularis* by qPCR in batches of approximately 300 pigs of the same age category with 95% confidence and an estimated prevalence of approximately 20% was calculated with the same software [35]. Data were collected in a spreadsheet program (Microsoft® Excel® Office Professional Plus 2016) and were then transferred to a statistic program (NCSS 12 Statistical Software (2018) NCSS, LLC. Kaysville, Utah, USA, ncss.com/software/ncss). Since in some countries more herds were sampled than planned, a random generator was used to include only 24 herds per country. A herd was defined as positive, if GE specific for *L. intracellularis* or corresponding antibodies were detected in at least one sample by qPCR and ELISA respectively. The apparent qPCR and ELISA herd prevalence was then calculated using the number of positive herds per country, divided by the total number of herds sampled per country. The apparent qPCR and ELISA within herd prevalence per country was calculated by the number of positive samples per herd, divided by the total number of samples analysed in this herd. Subsequently, the results of all herds per country were added and divided by the number of herds per country. The same procedure was used to calculate the detection rate per age category. The apparent prevalence was then converted to true prevalence using WinEpi [35]. According to the manuals of ‘SVANOVIR® L. intracellularis/ Ileitis-Ab’ from Boehringer Ingelheim SVANOVA®, Uppsala, Sweden, samples with a PI ≥30 were considered positive. All other samples, suspicious and negative samples were treated as negative. As part of the descriptive analyses the data was then tested for its` normal distribution using Shapiro- Wilk normality test. For further analyses, the qPCR and ELISA results were considering as dependent variable while age category and country were considered independent variables. Due to the fact that always more than two groups of continuous, not normally distributed variables were compared a ‘Kruskal- Wallis analysis of variance (ANOVA), Z test’ including Bonferroni correction was applied.

## Results

### Faecal samples: qPCR

Due to missing samples or insufficient sample material (less than 2 g faeces) in 30 cases, only 6450 samples of 144 herds were analysed and were included in the following results.

At least one positive sample per herd was found in 130 herds, which leads to an apparent prevalence of 90.3% while the true prevalence (90.3%) ranged from 85.4 to 95.1%. Of these herds, 85.4% had more than 3 positive samples. With a 100%, the UK reflects the highest percentage of positive herds, while the lowest percentage of positive herds was detected in France with 79.2% (Table 2). However, there was no statistical significant difference between the numbers of positive herds per country.

**Table 2:**
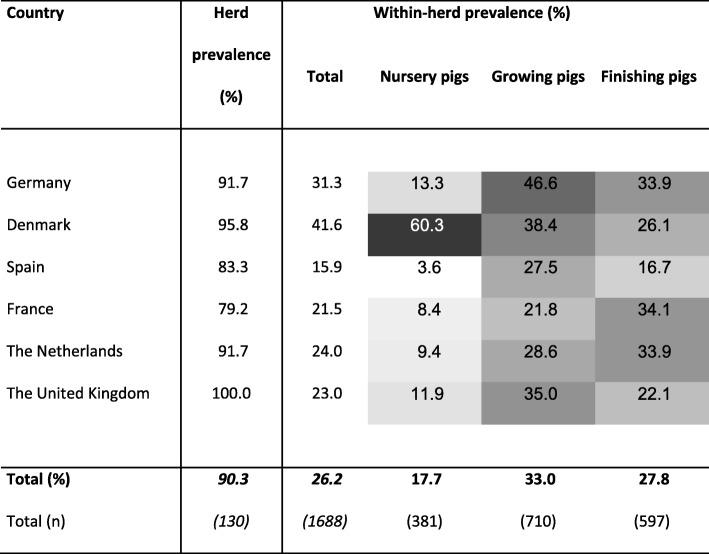
Herd prevalence and within-herd prevalence in total and per age category detected by qPCR.

A herd was defined as positive if genome equivalents were found in at least one sample per herd. The herd prevalence reflects the percentage of herds positive per country divided by the total number of herds sampled per country. The within-herd prevalence is the percentage of samples per herd and country with *L. intracellularis* detection. All measured by qPCR of 144 herds and 6450 faecal samples. The darker the field in the table, the higher the percentage of positive samples/ herds.

With 1688 positive faecal samples, a within- herd prevalence of 26.2% (Table 2) was calculated leading to a true prevalence of 26.67% (range: 13.8 to 39.6%). The number of positive faecal samples per positive herd in DK (median 18.0 min: 1 max: 42) was significant higher (*p* < 0.001) compared to UK (8.5 min: 1 max: 35) and ES (7.0 min: 1 max: 21), while no significant differences could be seen for DE (16.5 min: 2 max: 26), NL (11.0 min: 3 max: 28), or FR (10.0 min: 2 max: 28). Regarding the affected age categories, 381 (17.7%) samples of nursery pigs (NP), 710 (33.0%) of growing (GP) and 597 (27.8%) of finishing pigs (FP) were tested positive (Table 2). All three age categories were significantly different in the number of positive animals (*p* < 0.001), when compared to each other. NP were the rarest and GP the most frequently represented positive group. On country level this did not match for DK, were NP were significantly more often positive (*p* < 0.001) than Danish GP and FP. Moreover, significantly more NP per herd in DK were positive (median: 12 min: 0 max: 15) than in all other countries (NL 1 min: 0 max: 6, DE 0 min: 0 max: 14; UK 0 min: 0 max: 13, FR 0 min: 0 max: 10, ES 0 min: 0 max: 4; p < 0.001). The number of GP and FP positive per herd and country did not differentiate significantly.

#### Concentration of GE/ μl

In total, about half of the positive samples (50.3%) showed a concentration of 10^0^ GE/ μl. Concentrations of 10^4^ GE/ μl or higher could be detected in 7.7% of the positive samples. The percentage of samples with 10^4^ or more GE/ μl out of the overall positive samples per country was composed as follows: DK 13.4%, DE 6.8%, FR 6.6%, NL 5.8%, UK 5.7% and ES 1.7%. The concentration of GE/ μl detected in positive herds in DK (median: 26.6 min:1 max: 9.0 *×* 10^6^) was significantly higher (*p* < 0.001) compared to all other countries (DE: 12.6 min: 1 max. 1.5 *× 1*0^6^; ES:8.5 min:1 max: 4.8 *×* 10^4^; NL: 5.3 min: 1 max: 5.5 *×* 10^5^; UK: 4.6 min: 1 max: 8.4 *×* 10^5^; FR: 3.82 min: 1 max: 2.0 *×* 10^5^). While concentrations of 10^6^ GE/ μl were detected in four samples in Denmark and one sample in Germany, this concentration could not be detected in the other countries. Spain was the only country where also no samples with 10^5^ GE/ μl were found.

**Fig. 1:**
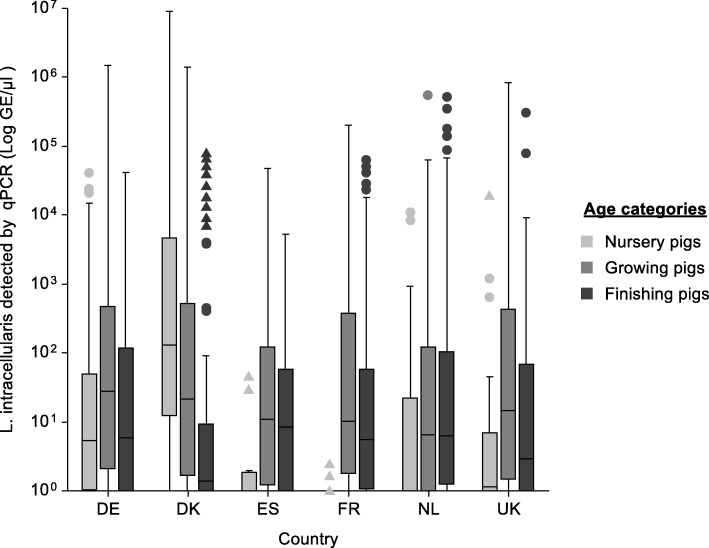
Concentration (Log GE/μl) of *L. intracellularis* in European countries by age categories. The Box plot was calculated from data of 1688 positive faecal samples of 130 herds in Germany (DE), Denmark (DK), Spain (ES), France (FR), the Netherlands (NL) and the United Kingdom (UK) all analysed by qPCR and separated by age category. Nursery pigs approx. 10 to 25 kg, growing pigs approx. 25 to 40 kg, finishing pigs approx. 40 to 100 kg. Mild outliers: dot (●); Severe outliers: triangle (▲); Inter- Quartile Range (IQR): Whiskers Boundaries Box Edge ±1.5 x IQR, Severe Quartile Boundaries: Box Edge ±3.0 x IQR

Comparing the age categories with each other, not including country as a stratum, positive FP shed significantly less GE/ μl (median 3.98 min: 1 max: 5 *×* 10^5^) than NP (18 min: 1 max: 9.0 *×* 10^6^) and GP (15.6 min: 1 max: 1.5 *×* 10^6^) (*p* < 0.001). However, if shedding is analysed on country level, this changes in all countries besides DK (Fig. 1). In DE, FP shed significantly less GE/ μl than GP (*p* < 0.003), while no difference was detected compared to NP. On the other hand, NP in ES (*p* < 0.003), FR (*p* < 0.001) and the NL (*p* < 0.02) shed less GE/ μl than the two other categories while in the UK this difference could only be seen between NP and GP (p < 0.001). The concentration of detected GE/ μl in NP in DK was significantly higher (*p* < 0.001) than in all other countries (Fig. 1). All together in DK the highest concentration of GE/ μl were shed in NP while in all other countries the GP tended to shed the highest amount. In addition, FP in DK excreted significantly less GE/ μl than FP in FR and NL (*p* < 0.03). No difference between the concentration of GE/ μl shed in GP was seen.

### ELISA

Due to missing or insufficient sampling material or wrong declaration in nine cases, only 1791 samples from 60 herds were analysed and are included in the following results.

#### Herd prevalence

In 91.7% of the sampled herds, antibodies against *L. intracellularis* were detected in at least one serum sample per herd (Table 3). The true seroprevalence (91.7%) ranged from 84.7 to 98.7%. The number of positive herds per country did not differ significantly. In five herds no antibodies against *L. intracellularis* were detected.

**Table 3:**
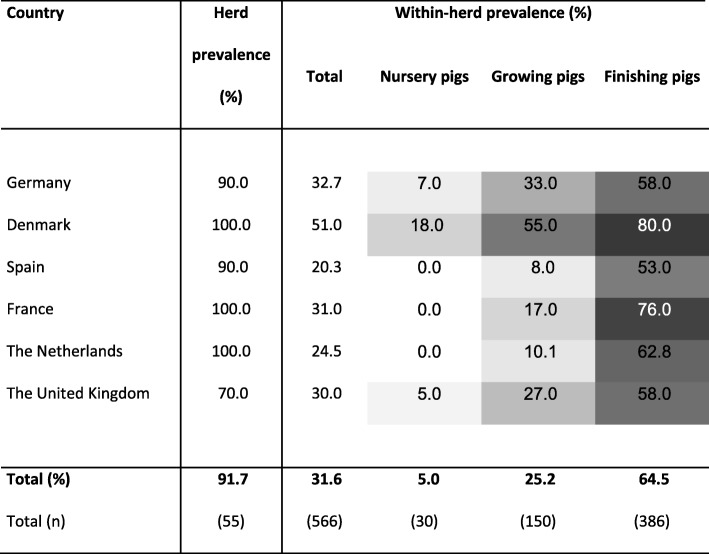
Herd prevalence and within-herd prevalence in total and per age category detected by ELISA.

A herd was defined as positive if antibodies were found in at least one sample per herd. The herd prevalence reflects the percentage of herds positive per country divided by the total number of herds sampled per country. The within-herd prevalence is the percentage of samples per herd and country with *L. intracellularis* antibody detection. All measured by ELISA of 60 herds and 1791 serum samples. The darker the field in the table, the higher the percentage of positive samples/ herds.

#### Within-herd prevalence

The average percentage of samples with antibodies against *L. intracellularis* within herds was 31.6% (Table 3) resulting in a true prevalence (33.3%) ranging from 9.48 to 57.19%. The number of positive samples per positive herds in DK was, with a median of 16.5 (min: 6 max: 24) significantly higher (*p* < 0.01) than in ES (8 min: 2 max: 10) and the NL (7.5 min: 1 max: 17). No significant difference was found for DE (10 min: 3 max: 27), FR (10.5 min: 1 max: 19) and the UK (11 min: 6 max: 24). Regarding the affected age categories, 30 (5.0%) samples of nursery pigs, 150 (25.2%) of growing and 386 (64.6%) of finishing pigs were positive for antibodies against *L. intracellularis* (Table 3). The number of positive samples per age category was increasing significantly (*p* < 0.001) from nursery to finishing pigs, while on country level, no significant difference between the age categories was observed.

From a total of 60 herds sampled for faeces and blood, only one herd was completely negative in both, qPCR and ELISA.

## Discussion

The sample size for this study was calculated for a within- herd prevalence of at least 20% detected by qPCR. In ES, FR, the NL and the UK this fits quite well while in DE and especially DK significantly more samples were positive. Therefore the detection of the disease in these two countries would have been possible with even less samples per age category. In order to calculate the number of required herds, a prevalence of 50% was assumed and an error of 20% was accepted. Since the herd prevalence, detected in this study was clearly higher, the confidence interval is reduced, and the error is smaller. Nevertheless, the number of herds tested for blood per country was low, causing a high range of the true seroprevalence and even higher range regarding the true within- herd seroprevalence. Although a 100% of the farms in DK, FR and the NL in this study were seropositive, it can’t be assumed, that this is also the case for every other farm in one of these countries. From a statistical point of view, such a statement of 100% positive herds without sampling all herds in the country is anyway not permissible. The ELISA results are therefore rather ‘detection rates’ than ‘prevalence’.

Nevertheless, the apparent and true prevalence detected by qPCR and ELISA, as well as the fact that more than 85% of positive herds in qPCR had more than 3 samples positive, underline the fact, that *L. intracellularis* is widespread in European pig herds. This is consistent with reported prevalence in other continents [1–4].

In general, the prevalence detected in this study is slightly higher compared to literature (Table 1), taking the age category and the diagnostic test used into account [12, 16].

Especially the within-herd prevalence determined by qPCR was higher than described in publications from DE and DK [9, 11, 13]. This could be caused by the given inclusion criteria of at least one outbreak of diarrhoea in the preceding 12 months before the date of the examination. Therefore, the available results cannot be applied to the entire pig population of a country. In addition, the proportion of completely free herds per country might therefore be underestimated. Furthermore, it was the responsibility of the veterinarians sampling the herds, to determine which herds were selected, as long as exclusion criteria were met. An equally geographical distribution in the countries can therefore not be guaranteed, whereby at least in Germany no indications of differences in detection rate between north and south exist [12, 30]. Another reason for a higher prevalence detected by qPCR in this study could be a reduced use of antimicrobials, recently and in the last years. It is known that the administration of antibiotics reduces the determined quantity of *L. intracellularis* [36–38] and thereby reduces the detection rate in PCR. However antibiotics, as growth promoters in animal feed, have been banned in the EU since 1 January 2006 [39]. This has led to a reduction in the amount of antimicrobials used in farm animal production in Europe [40]. In addition, antimicrobial usage up to four weeks before sampling, was an exclusion criterion in the present study in contrast to the studies listed in Table 1. Negative influences of antimicrobial treatments on seroconversion are discussed. A potential delay or lack in seroconversion is assumed [2, 37, 38, 41]. However, it is uncertain whether this influenced the detection rates in the present study, or not.

Other possible reasons for higher detection rates in this study could be a stronger spread of the pathogen over time as well as potentially different sensitivity and specificity of the performed tests (PCR: conventional < nested < real-time; serology: ELISA >IFA > IFAT) [42].

The only country where fewer herds were positive than described in literature (Table 1) was the United Kingdom. While in a study from 2010 [14] 93.1% of the herds in UK were seropositive using indirect immunofluorescence assay, antibodies in the present study were only detected in 70% of the herds. A reduction of the overall percentage of seropositive pigs in British and Irish herds has already been reported from 1998 to 2008 [14]. The authors explain this with the inclusion of fewer single-site farms in 2008 than in 1998 and possibly improved hygiene and improved control measures. However, GE in the present study were detected in a 100% of the herds with no significant differences in the concentration of GE/ μ detected compared to other countries. A study in Croatia describes a faster decrease and less seropositivity in animals kept outdoors [43]. However, this could be due to lower stocking density, reduced stress and a more natural intestinal microbiota [31]. Based on data of the GOV-UK-annual statistics about agriculture in the United Kingdom, land used for outdoor pigs increased from 8000 (2014) to 11,000 ha (2018), which probably led to an increase in the number of pigs kept outdoors. Contrariwise, significant differences in the within-herd prevalence or the age categories, compared to other countries, as would be expected with a faster decrease in antibodies, were not observed.

Information regarding the current European available live vaccine report no excretion of the vaccine strain [44]. Whether the use of a qPCR with higher sensitivity and specificity leads to the detection of lyophilized vaccine bacteria cannot be clarified here. With regard to the excretion after challenge, studies differed in quantity and duration of *L. intracellularis* shedding [19, 36, 44, 45]. As vaccination was no exclusion criteria, vaccination should be considered a potential influencing factor on the concentration of GE/ μl and number of positive samples found. Since the current European available live vaccine does not induce antibody production [44], it can be assumed that seroconversion of animals in this study was due to infections with the wild type of *L. intracellularis*. Since the youngest participating group in this study were NP with an approximate weight of 10 to 25 kg, even the presence of maternal antibodies is relatively unlikely [10, 24, 28, 29].

NP in the present study were generally significantly less often positive than the other two age categories in both, qPCR and ELISA which is described in literature as well [2, 12, 30, 46].

Furthermore, the early shedding of Lawsonia, as observed in this study in DK, has also already been described in a Danish study [23]. In contrast to the recently mentioned study and other publications [21–23], GE of *L. intracellularis* in the present study could also be detected in animals at slaughter. However, the exact age of the animals in the present study has not been recorded. It can therefore not be proven, if animals from an age of 14 to 18 weeks were still shedding *L. intracellularis*.

Regarding the discussion about the persistence of antibodies [19, 21–23], in the present study the number of animals with antibodies until slaughter has increased continuously and significantly. Whether the animals were undergoing re-infection/ booster, or whether antibodies naturally persist for a longer time, was not examined.

DK was the country with the highest determined quantity of GE among NP. In all other countries shedding was seen later in time in GP. A tendency of increased antibody presence in Danish NP compared to NP of the other countries was seen and also fits to a recent publication from DK [23] and to the known antibody formation about two weeks after infection [23, 24]. Due to uniform EU regulations regarding weaning (COUNCIL DIRECTIVE 2008/120/EC Annex I, Chapter II, C. Piglets), maternal antibodies should not lead to elevated antibody titres in only one country. Conclusively an earlier contact with the pathogen in DK, compared to the other European countries can be assumed. As shedding is described already one week after exposure [19, 20], contact could probably be at the end of the suckling period or beginning of weaning. As maternal antibodies in this time drop [10, 24, 28, 29] and protective action of maternal antibodies in general are discussed [24, 30], NP might well be susceptible for the pathogen at this time.

If earlier contact would be the case, the question arises if Danish animals develop an earlier protective immunity [32] than animals in the other countries. As a logical conclusion, the number of animals with faecal shedding and/or the concentration of excreted and determined quantity of *L. intracellularis* in animals with protective immunity should be lower compared to animals without or with only partly protection. In contrast to this hypothesis, no differences were found in the number of positive GP or FP detected by qPCR or ELISA between the countries. The concentration of GE/ μl detected in faeces of GP did not vary from country to country. On the other hand, DK was the only country with a lower determined quantity of GE in FP compared to both other age categories. Significantly less GE shedding of FP was seen compared to French or Dutch FP. Overall FP in DK may already process or build up immunity earlier and thus reduce the amount of excreted *L. intracellularis*. However, a final statement on this would have to be made in a longitudinal rather than in a cross-sectional study.

NP in DK shed GE in concentrations of 10^0^ to 9.0 × 10^6^ GE/ μl. They almost reached the upper limit of quantification of the qPCR. Such high values were much less frequently or not at all achieved in the other countries. It is speculative whether this is due to the fact that animals in other countries become diseased later in age and the immune system then reacts more effective to the infection, or shedding is higher due to another unknown reason. The high concentration of determined GE from NP also led DK in total to differ significantly in the concentration of determined GE from the other countries.

10^0^ GE/ μl is the lower limit of detection of the qPCR used in this study. How these results should be interpreted, is not described in the manual of the mentioned commercial kit. Since the presence of the pathogen does not necessarily lead to disease [17] and due to the ubiquitous presence [18] of the pathogen and the correlation between excreted dose and histopathological lesions [26, 27], it was assumed that animals with 10^0^ GE/ μl do not suffer from an acute infection. Values of 10^4^ GE/ μl or higher were found only sporadically, which leads to the conclusion that the pathogen has proliferated in the intestines of the animals. Therefore, concentration of 10^4^ GE/ μl or more were considered as high. However, it must be taken into account, that no pathohistological examinations of the animals were carried out and that the average daily gain evaluations are not performed yet. Therefore, 10^4^ GE/ μl is not to be seen as a threshold value but as an interpretation of the data in that situation. However, due to the intermittent excretion of *L. intracellularis* [19, 22], not all animals may shed *L. intracellularis* in high concentration at the same time. Therefore, it is the opinion of the authors, that if high concentrations are detected in single samples, whereby the majority of the rest of the samples show low or no GE, an infection of the herd with potential economic consequences is nevertheless likely.

## Conclusion

A widespread of *L. intracellularis* in the six European countries was confirmed, whereby a large part of the positive animals only excreted small concentration of GE and the economic impact of these are currently unknown. Although the herd prevalence did not differ significantly by qPCR or ELISA, significant differences between countries regarding within-herd prevalence, affected age categories, number of positive animals and concentration of GE shed were observed. Nevertheless, qPCR provides more valuable information regarding infection and is therefore for this question preferable to non-quantitative methods. Infections with *L. intracellularis* seem to vary between different countries. For a more detailed evaluation of potential risk factors, a further report will highlight the results of the analyses of the epidemiological data collected in this study, and hopefully give additional information about the reasons for the country specific differences found in this investigation.

## Data Availability

The datasets used and analysed during the current study are available from the corresponding author on reasonable request.

## Abbreviations

DE: Germany
DK: Denmark
ELISA: Enzyme linked immunosorbent essay
ES: Spain
FP: Finishing pigs
FR: France
GE: Genome equivalents
GP: Growing pigs
L. intracellularis: Lawsonia intracellularis
NL: Netherlands
NP: Nursery pigs
qPCR: Quantitative polymerase chain reaction
UK: United Kingdom
